# Depletion of CG-Specific Methylation in *Mycoplasma hyorhinis* Genomic DNA after Host Cell Invasion

**DOI:** 10.1371/journal.pone.0142529

**Published:** 2015-11-06

**Authors:** Andrei V. Chernov, Leticia Reyes, Scott Peterson, Alex Y. Strongin

**Affiliations:** 1 Infectious & Inflammatory Disease Center, Sanford Burnham Prebys Medical Discovery Institute, La Jolla, California, United States of America; 2 Department of Infectious Disease & Pathology, College of Veterinary Medicine, University of Florida, Gainesville, Florida, United States of America; The University of Melbourne, AUSTRALIA

## Abstract

Adaptation to the environment requires pathogenic bacteria to alter their gene expression in order to increase long-term survival in the host. Here, we present the first experimental evidence that bacterial DNA methylation affects the intracellular survival of pathogenic *Mycoplasma hyorhinis*. Using bisulfite sequencing, we identified that the *M*. *hyorhinis* DNA methylation landscape was distinct in free-living *M*. *hyorhinis* relative to the internalized bacteria surviving in the infected human cells. We determined that genomic GATC sites were consistently highly methylated in the bacterial chromosome suggesting that the bacterial GATC-specific 5-methylcytosine DNA methyltransferase was fully functional both pre- and post-infection. In contrast, only the low CG methylation pattern was observed in the mycoplasma genome in the infective bacteria that invaded and then survived in the host cells. In turn, two distinct populations, with either high or low CG methylation, were detected in the *M*. *hyorhinis* cultures continually grown in the rich medium independently of host cells. We also identified that *M*. *hyorhinis* efficiently evaded endosomal degradation and uses exocytosis to exit infected human cells enabling re-infection of additional cells. The well-orchestrated changes in the chromosome methylation landscape play a major regulatory role in the mycoplasma life cycle.

## Introduction

DNA methylation is one of a few major epigenetic mechanisms that regulate gene expression. In eukaryotes, DNA methylation—the conversion of cytosine to 5-methylcytosine (5mC) in the context of CG-dinucleotides—is catalyzed by DNMT1, DNMT3A and DNMT3B 5mC-DNA methyltransferases (MTases). On average, CG dinucleotides are underrepresented in the human genome compared to other six dinucleotide combinations. A higher than average number of CGs is observed within CpG islands (CPGIs). CPGIs are typically associated with the gene promoter regions [1]. Aberrant global and gene-specific, DNA hypo- and hypermethylation is frequently reported in multiple cancer types [2].

In prokaryotes, DNA methylation is involved in restriction-modification system that protects bacteria from bacteriophages. Methylation of cytosines and adenosines occurs within or near the sequence-specific DNA recognition sites. Adaptation to multiple distinct environments requires pathogenic bacteria to alter their gene expression to increase fitness and long-term survival in the host. The roles of DNA methylation during pathogenesis are poorly described but have recently been recognized in several important pathogens [3, 4]. Here, we present the first experimental evidence that bacterial DNA methylation affects the intracellular survival of pathogenic *Mycoplasma hyorhinis*.

Mycoplasmas (class *Mollicutes*) are the smallest parasitic self-replicating microorganisms and in nature depend on host cells for survival. Mycoplasmas colonize and invade both animal and human cells and exhibit the ability to evade the immune defense and antibiotic treatment [5–8]. In humans, mycoplasmas frequently populate mucosal surfaces, often persist as long-term asymptomatic infections and likely promote chronic aberrant states in the infected tissue [6]. Although a direct role of mycoplasmas, including *M*. *hyorhinis*, remains controversial in carcinogenesis, evidence of the common presence of mycoplasmas in the prostate, renal, gastric, colon, esophageal, lung, breast, ovarian and melanoma tumors suggests, at least, the favorable co-existence of mycoplasmas and tumors [9–14]. *M*. *hyorhinis* is also notoriously known as cell culture contaminant. Intriguingly, invasive bacteria, including mycoplasmas and mycobacteria, can induce reprogramming of somatic cells [15] and deregulate cancer-specific genes, including RAS and MYC oncogenes, and the p53 tumor suppressor [12, 16–18] leading, ultimately, to oncogenic transformation.

Recently, we cloned and characterized three *M*. *hyorhinis* MTases (Mhy1, Mhy2 and Mhy3) with the 5mC methylation activity, and the CG (Mhy1 and Mhy2) and GATC (Mhy3) substrate specificity [19]. Upon their expression in human cells, all three MTases readily translocated into the host cell nucleus. In the nuclei, the bacterial enzymes effectively methylated their respective recognition sites in the human genomic DNA. However, it is not yet known whether mycoplasma MTases act in a similar fashion *in vivo* and whether they are capable of introducing aberrant methylation in the human genomic DNA in human cells infected by mycoplasma. To shed light on the functionality of the *M*. *hyorhinis* MTases *in vivo* and to determine if the bacterial Mhy1, Mhy2 and Mhy3 enzymes are active in both free living mycoplasma and mycoplasma surviving inside the host cells, we determined the methylation landscape of the *M*. *hyorhinis* genome in the course of bacterial infection of human host cells.

## Materials and Methods

### Reagents

Reagents were obtained from Fisher Thermo Scientific (Waltman, MA) unless otherwise indicated. The antibodies we used were murine monoclonal antibodies to *M*. *hyorhinis* P70 protein (clone AB3P, a gift from K. S. Wise, University of Missouri, Columbia, MO), RAB5 (Abcam, Cambridge, MA), RAB7 (Santa Cruz Biotechnology, Dallas, TX) and LC3 (Abgent, Atlanta, GA), a mule antiserum to *M*. *hyorhinis* (a gift from M. B. Brown, University of Florida, Gainesville, FL), a rabbit monoclonal antibody to RALA and a rabbit polyclonal antibody to EXOC7 (GeneTex, Irvine, CA). Goat anti-rabbit AlexaFluor 488 and 647, goat anti-mouse AlexaFluor 488 and 594 (Life Technologies, Grand Island, NY) and rabbit anti-donkey Dylight-594 (Bethyl Laboratories, Montgomery, TX) were used as the secondary antibodies.

### Cell culture

Cell culture media were from Life Technologies unless otherwise indicated. Cell cultures were maintained at 37°C and 5% CO_2_. Human transformed first trimester extravillous HTR8/SVNeo trophoblasts [20, 21] were grown in RPMI-1640 medium supplemented with 5% FBS and 100 μg/ml primocin (Invivogen). Primocin was not included in the medium samples used for infecting host cell with mycoplasma.

### Cultivation of *M*. *hyorhinis* and infection of human cells

*M*. *hyorhinis* strain SK76 was kindly provided by Kim S. Wise (University of Missouri, Columbia, MO) [22]. Planktonic mycoplasma was grown in the SP4 glucose broth (Remel, Lenexa, KS) and Frey’s agar [23]. For infection, a frozen (-80°C) *M*. *hyorhinis* aliquot was diluted in 10 ml broth and incubated for 16 h at 37°C. The sensitivity of *M*. *hyorhinis* to gentamicin was established as described by others [5]. Briefly, bacteria (1x10^6^ colony-forming units) were inoculated into the broth supplemented with gentamicin (300 μg/ml). An antibiotic-free culture was used as a control. In 48 h, medium aliquots were plated onto agar plates and incubation was continued at 37°C. Emerging *M*. *hyorhinis* colonies were then counted [5]. In contrast to multiple mycoplasma colonies in control samples, there were no surviving mycoplasmas in the antibiotic-treated samples.

HTR8/SVneo trophoblasts (1x10^5^ cells per infection, N = 3) were seeded in RPMI supplemented with 10% fetal bovine serum (RPMI/FBS) and incubated for 24 h. Trophoblasts were infected at a multiplicity of infection of 100 using *M*. *hyorhinis* diluted in RPMI/FBS. In 2 h post-infection, cells were washed in PBS. The washed cells were then transferred into RPMI/FBS supplemented with gentamicin (300 μg/ml). Incubation was continued for an additional 1, 4 or 22 h (these timing corresponded to 3, 6 and 24 h post-infection). Long-term infections were conducted by culturing trophoblasts in RPMI/FBS substituted with gentamicin (300 μg/ml) for a total duration of 3–14 days.

### Bisulfite sequencing

Genomic DNA was purified from the cultured, planktonic and the intracellular *M*. *hyorhinis* cells that colonized the host cells, using the genomic DNA purification system (Zymo Research, Irvine, CA). The bisulfite DNA conversion was performed using an EZ DNA Methylation-Lightning system (Zymo Research). DNA fragments were amplified by PCR in 50 μl reactions using EpiMark Hot Start Taq DNA Polymerase (New England Biolabs, Ipswich, MA), dNTPs (200 μM each), bisulfite converted DNA template (10–50 ng), and the forward (5’-TGTGGTTGATTGTAAGATTTATAAGT-3’) and reverse (5’-TTAACAAAACAACTAAAACACCAAC-3’) primers (0.5 μM each). PCR products were purified, inserted into the pMiniT vector (New England Biolabs) and transformed into 10-beta *E*. *coli* cells. Individual positive *E*. *coli* clones were randomly picked and the DNA inserts in these clones were then sequenced.

### *In situ* immunofluorescent confocal microscopy

Cells were seeded in wells of a Lab-Tek II CC2 glass chamber and grown to reach a 70% level of confluence. Cells were fixed for 10 min in 4% para-formaldehyde at 25°C, permeabilized for 5 min in 0.25% Triton X-100, and blocked for 1 h in 2% BSA at 25°C. Cells were incubated for 1 h with the respective primary antibodies followed by incubation (30 min; 25°C) with species-specific secondary antibody. Cells were mounted on slides using the Prolong Gold reagent (Life Technologies) containing 4',6-diamidino-2-phenylindole (DAPI). Confocal microscopy was conducted using a LSM 710 NLO Zeiss Multiphoton Laser Point scanning confocal microscope equipped with a multi-photon Mai-Tai laser HB–DeepSee system (690–1024 nm). Images were acquired using ZEN software (Zeiss, Thornwood, NY). Alternatively, a Spinning Disc confocal microscope DSU-IX81 (Olympus, Waltham, MA) was used and images were captured using Slidebook software (Olympus). Images were further processed using ImageJ (http://www.macbiophotonics.ca). Our experiments were performed multiple times and at least three images were obtained from three independent biological replicates. Colocalization analysis was conducted as previously described [24]. The data were analyzed using Statview software (SAS Institute, Cary, NC; http://www.statview.com). The p-values below 0.05 were considered significant.

### *M*. *hyorhinis* transmission assay

Cell Tracker Green CMFDA reagent (Life Technologies) was used to prepare the fluorescently labeled HTR8/SVneo cells. For primary infection performed in triplicates, unlabeled HTR8/SVneo cells were infected with *M*. *hyorhinis* as described above. At 2 h post-infection, cells were treated with gentamicin (300 μg/ml). In 4 h, extracellular mycoplasma was removed by multiple washings. Uninfected Tracker Green-labeled naive HTR8/SVneo cells were added to the infected cell samples. Incubation was continued for an additional 24 h in antibiotic-free RPMI/FBS. Cells were fixed and *M*. *hyorhinis* was visualized using the P70 antibody.

### DNA sequence analysis

Genomic DNA sequence of *M*. *hyorhinis* SK76 strain [22] was obtained from ftp.ncbi.nih.gov/genomes/bacteria. Dinucleotide analysis of *M*. *hyorhinis* genome sites was performed using our custom Perl scripts. CG-dense regions were identified using CpGcluster software [25]. Mapping of *M*. *hyorhinis* genome and genome representation in a circular diagram form was performed using Circos software [26].

## Results

### *M*. *hyorhinis* invades human cells and associates with the exocysts

Although invasion of human cells by infective mycoplasma has been widely observed, the precise mechanisms of host cell invasion and post-infection compartmentalization remain insufficiently understood. To identify cellular compartments occupied by invasive *M*. *hyorhinis*, we analyzed the post-infection events in more detail. We used human trophoblast cells in our study as a surrogate model of mycoplasma infection. Thus, HTR8/SVneo trophoblasts were infected with *M*. *hyorhinis* and cultivated for an additional 2 h. To eradicate non-internalized mycoplasma, trophoblasts were incubated in medium supplemented with gentamicin.

**Fig 1:**
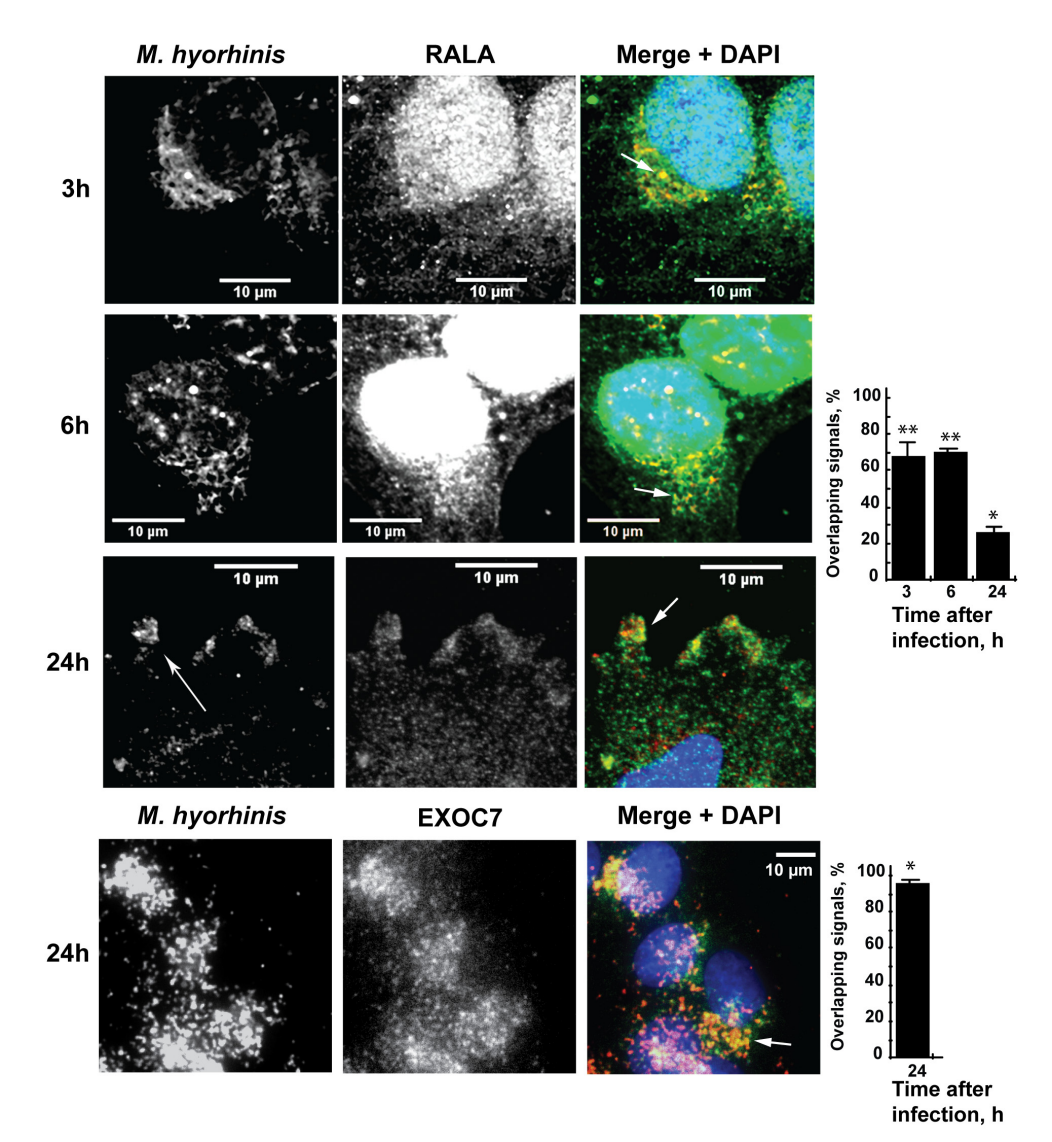
Confocal microscopy of the immunoreactivity of *M*. *hyorhinis* with the RALA and EXOC7 exocytosis-specific markers in mycoplasma-infected HTR8/SVneo trophoblasts. In the RALA panels, the bars show the percentage of the mycoplasma signal overlapping with the RALA immunoreactivity at 3, 6 and 24 h post-infection (N = 3). * and **, the p-values below 0.05 and 0.005, respectively. In the EXOC7 panel, the bar shows the percentage of the mycoplasma signal overlapping with EXOC7 at 24 h post-infection. Arrows indicate overlapping signals. Scale bars, 10 μm. Blue, DAPI.

**Fig 2:**
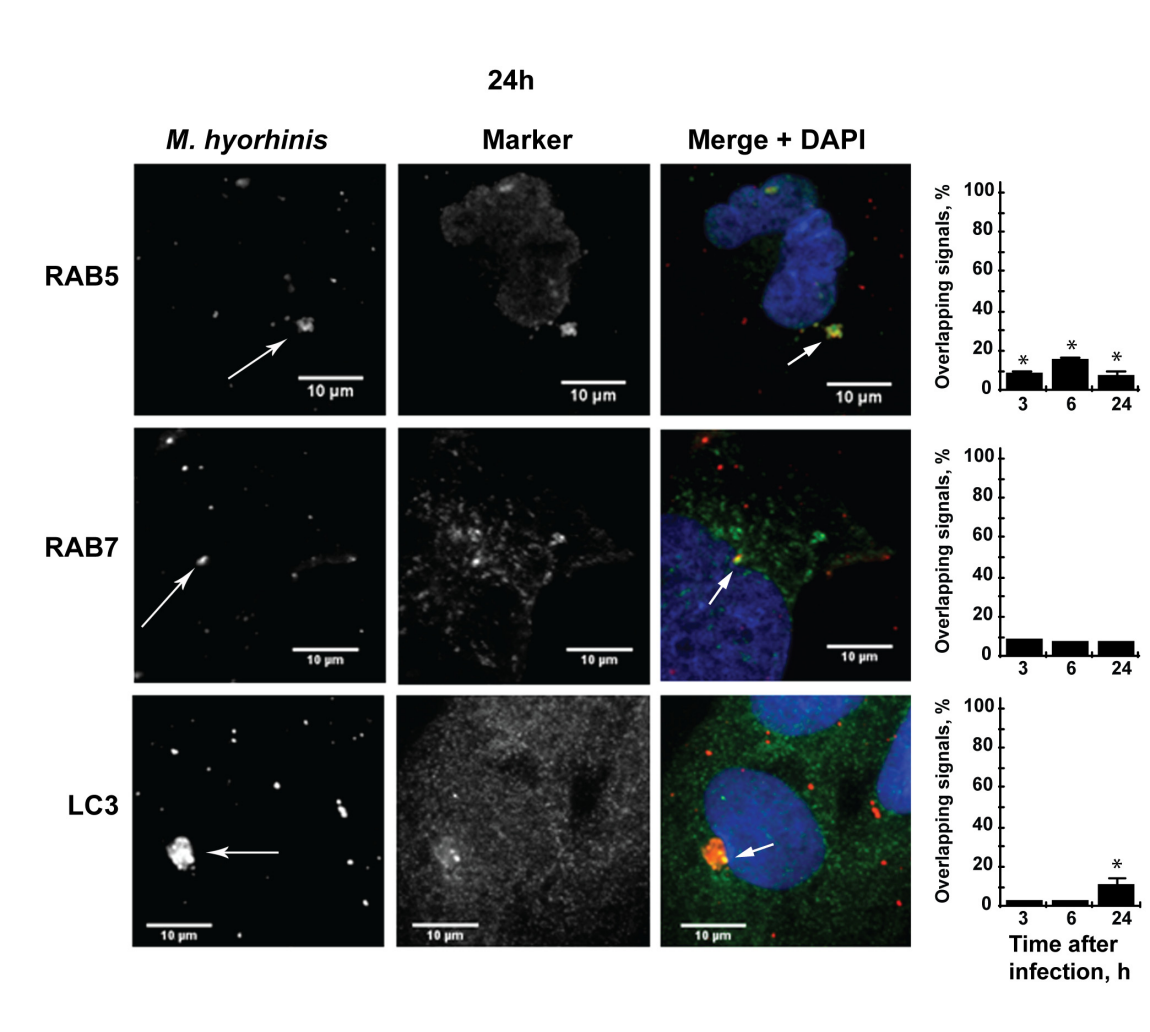
Confocal microscopy of the immunoreactivity of *M*. *hyorhinis* with the endosome-specific markers RAB5 and RAB7, and the autophagosome-specific marker LC3 in mycoplasma-infected HTR8/SVneo trophoblasts 24 h post-infection. In the RAB5, RAB7 and LC3 panels, the bars show the percentage of the mycoplasma signal overlapping with the corresponding markers at 3, 6 and 24 h post-infection (N = 3). *, the p-values below 0.05. Arrows point to the overlapping signals. Scale bars, 10 μm. Blue, DAPI.

The 3, 6 and 24 h post-infection samples were stained using the antibody to the *M*. *hyorhinis* P70 protein and to the early endosome marker RAB5, the late endosome marker RAB7, the exocytosis marker RALA and the autophagosome marker LC3. The highly specific P70 monoclonal antibody does not cross-react with other mycoplasma species and host cell proteins (http://www.ibridgenetwork.org/mu/antibody-against-p70-surface-antigen-of-m-hyorhinis-clone-ab3). Strikingly, there was a significant level of co-localization of the mycoplasma immunoreactivity with the exocytosis marker RALA, a GTP-binding protein that interacts with the exocyst complex (Fig 1) [27]. The co-localization of the intracellular mycoplasma with RALA was most noticeable at 3 h and 6 h post-infection (67%±14% and 68%±3%, respectively). The level of co-localization decreased to 27%±6% at 24 h post-infection. Furthermore, we consistently observed co-localization (95%±4%) of the intracellular mycoplasma with EXOC7, a subunit of the exocyst protein complex (Fig 1). In contrast, there was an insignificant level of co-localization of *M*. *hyorhinis* with both the endosomal (RAB5 and RAB7) and autophagosomal (LC3) markers in the infected trophoblasts (Fig 2). Based on our results, we suggested that the majority of the intracellular mycoplasma escapes cellular autophagocytosis and endosomal trafficking, and, instead, associate with exocysts.

### Viability of intracellular mycoplasma

**Fig 3:**
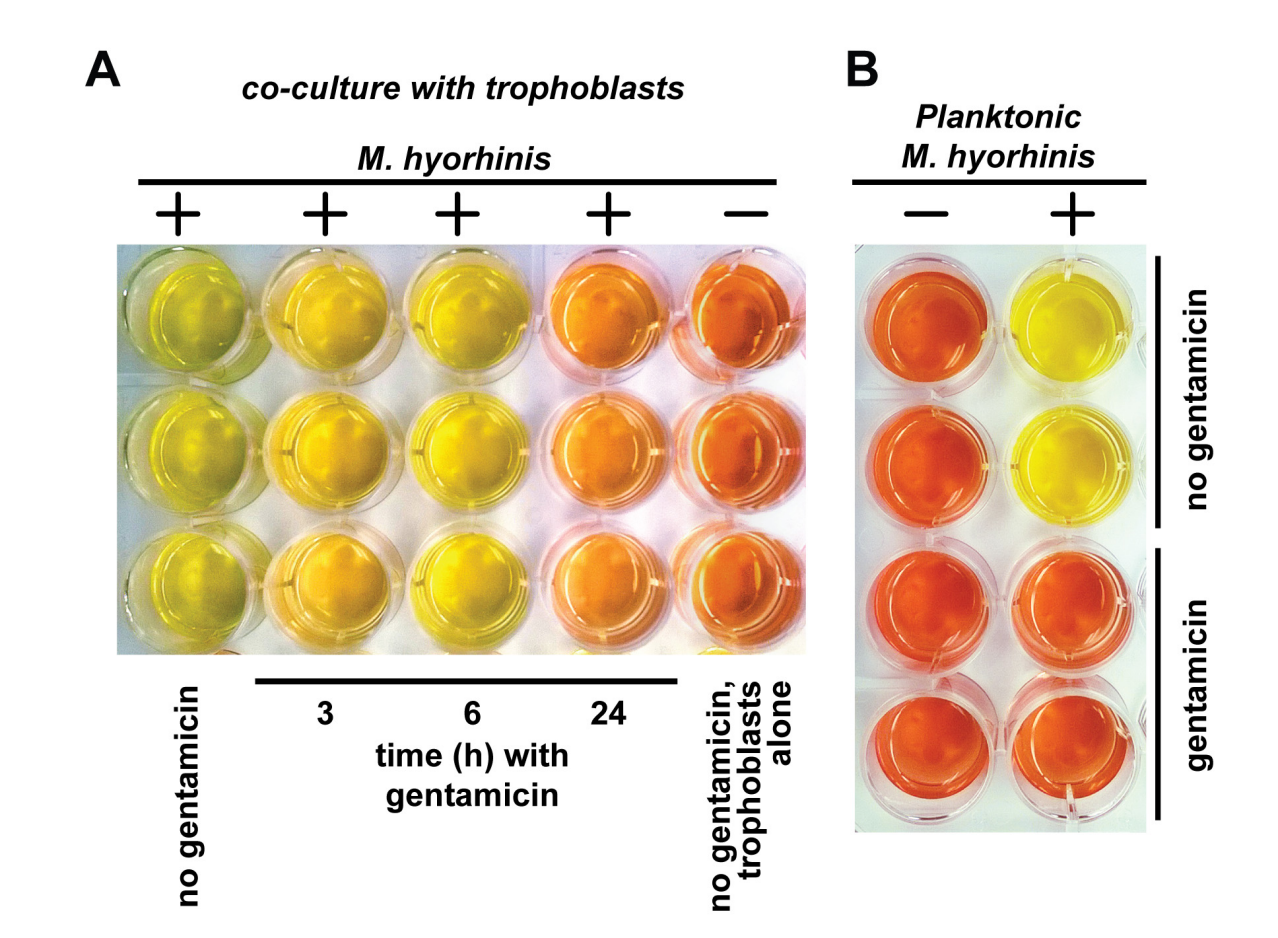
Viability of intracellular *M*. *hyorhinis*. A, Secondary growth of *M*. *hyorhinis* released from infected HTR8/SVneo trophoblast cells following gentamicin treatment (N = 3). Trophoblasts were infected with *M*. *hyorhinis* and co-incubated with gentamicin for 3, 6 and 24 h. The infected trophoblasts were washed in PBS. Then, 1 ml of the fresh SP4 broth was added to the samples. The samples were further incubated for 7 days to allow the secondary growth of mycoplasma. The secondary growth of planktonic mycoplasma was visualized by the changes in the color of the medium from orange-brown to yellow. B, The effect of gentamicin (300 μg/ml) on planktonic *M*. *hyorhinis* (N = 2) alone.

We hypothesized that the internalized mycoplasma remains viable and retains infectious capacity. To test this hypothesis, we infected HTR8/SVneo trophoblasts with *M*. *hyorhinis* followed by eradicating free-living, planktonic mycoplasma using gentamicin. Next, the medium was changed to the SP4 broth to detect secondary growth of planktonic mycoplasma. We observed a noticeable growth of planktonic mycoplasma in co-cultures exposed to gentamicin for 3 and 6 h (Fig 3A). However, a long exposure to gentamicin (24 h) inhibited the secondary growth of planktonic mycoplasma. We concluded that in the absence of host cells mycoplasma was highly sensitive to gentamicin and lacked the secondary growth (Fig 3B). We speculated that the release of intracellular mycoplasma into the broth and re-infection of host cells promoted the secondary growth.

**Fig 4:**
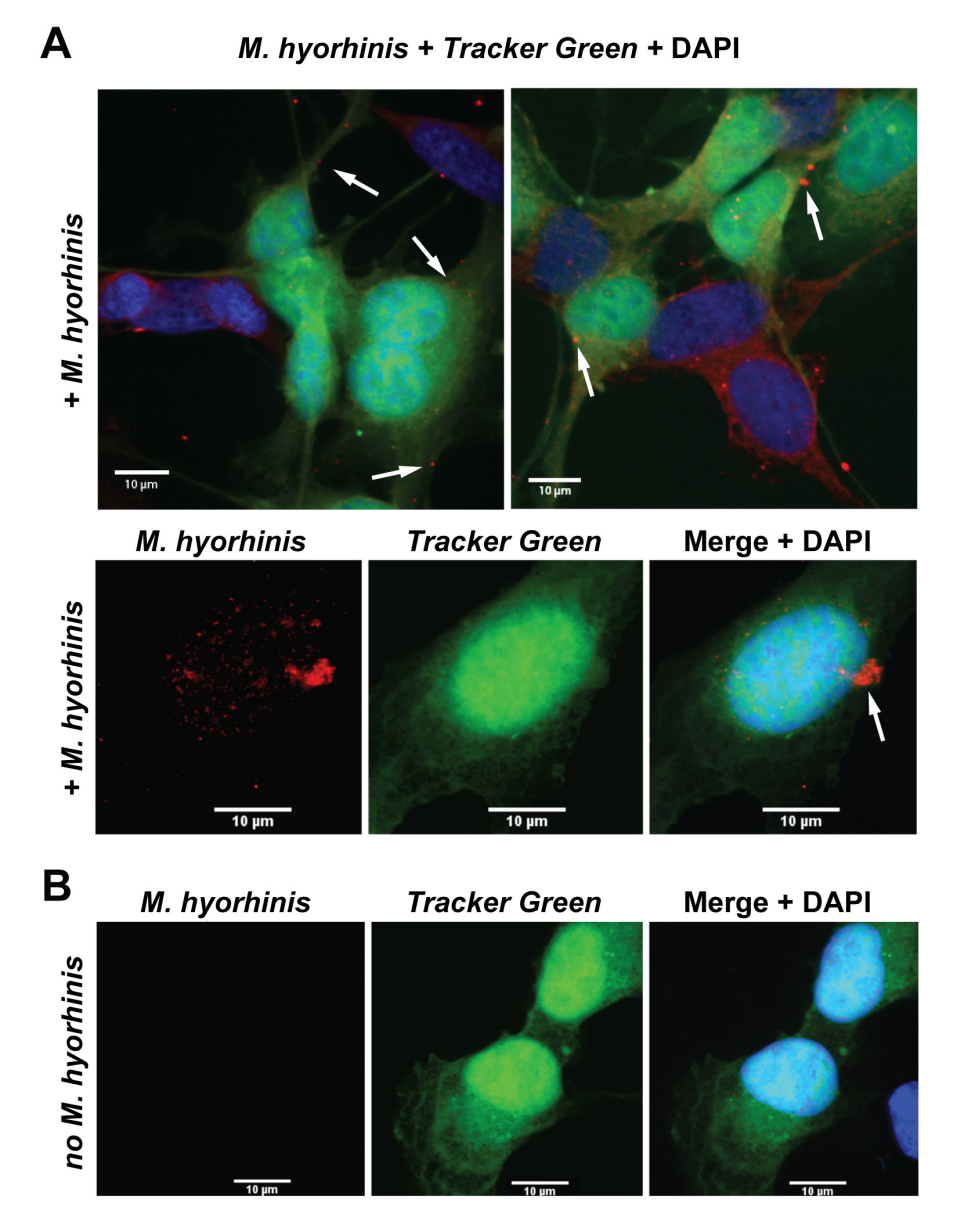
*M*. *hyorhinis* transmission assay. A, Confocal microscopy of HTR8/SVneo trophoblasts infected with *M*. *hyorhinis* (red) jointly with the naïve, fluorescently labeled trophoblasts (green). Top panel, HTR8/SVneo trophoblasts were infected for 2 h with *M*. *hyorhinis*. Gentamicin (300 μg/ml) was added to the samples for an additional 4 h. Then gentamicin was removed and the naive fluorescently labeled trophoblasts were added to the samples. After 24 h, the secondary infection of the naïve trophoblasts was recorded. The arrows point to the sites of the secondary infection in the fluorescently labeled trophoblasts. B, The non-merged images of the fluorescently labeled trophoblast (Tracker Green) infected by *M*. *hyorhinis* (red). DAPI, blue. Bottom panels, uninfected trophoblast cell control.

To further investigate infectious characteristics of the internalized *M*. *hyorhinis*, we studied whether the bacteria can re-infect the naive host cells. For these purposes, we co-incubated HTR8/SVneo cells with *M*. *hyorhinis* and then eradicated planktonic mycoplasmas using gentamicin. Naive, fluorescently labeled HTR8/SVneo cells were then added to the infected cultures. The samples were co-incubated further in the gentamicin-free medium to allow re-infection of the newly added cells by the internalized and then released mycoplasmas. We observed that the fluorescently labeled fresh cells readily acquired mycoplasma (Fig 4). These results indicated that the cell internalization did not abolish the ability *M*. *hyorhinis* to exit from the infected cells and then to re-infect the additional host cells.

### Mycoplasma DNA methylation in the free-living and intracellular *M*. *hyorhinis*

**Fig 5:**
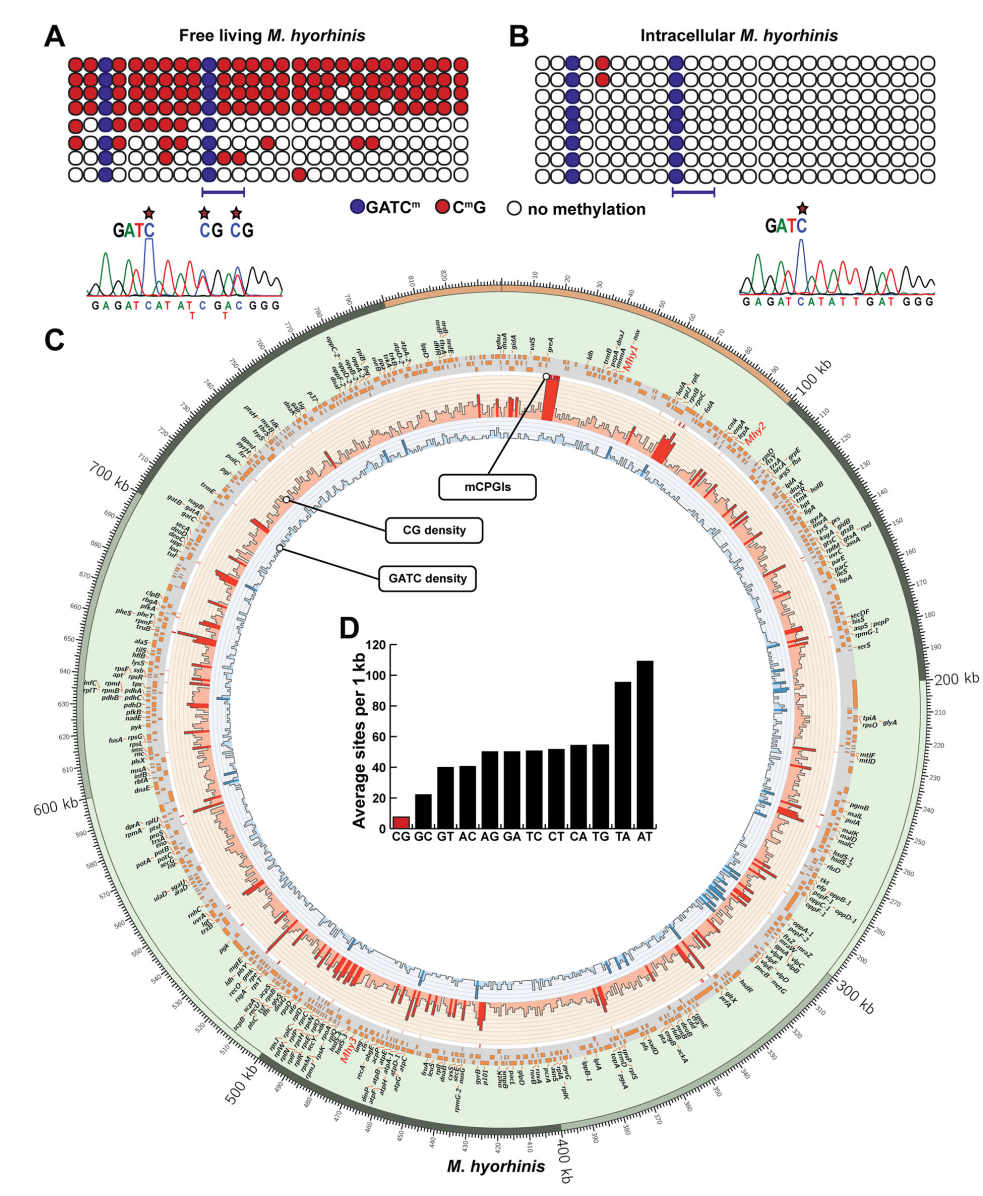
Methylation of *M*. *hyorhinis* genomic DNA. A and B, schematic diagrams show the presence of two GATC and 27 CG methylation sites in the 368 bp CG-rich region of the *M*. *hyorhinis* genome in free-living (A) and intracellular (B) mycoplasmas. Circles indicate methylated CGs and GATC sites (red and blue, respectively), and unmethylated (empty) sites. Each row corresponds to a methylation pattern in the individual mycoplasma cell. Bottom, a fragment of the sequencing chromatogram of the bisulfate-converted DNA confirms the presence of the methylated GATC and CG sites in the 368 bp CG-rich region of the *M*. *hyorhinis* genome. Stars, 5mC. C, Circular histogram shows the distribution of the CG and GATC sites in the *M*. *hyorhinis* genome. Histogram bar heights correspond to a number of the individual CG and GATC marks (light red and light blue, respectively) within a 1 kb window. Regions with >10 sites are shown in dark red (CG) and dark blue (GATC). Genes are named and their positions are shown along the chromosome as orange bars. In the genomic DNA, positions of mycoplasma CPGI-like regions (mCPGIs) are indicated by thin red lines on the white background. The histogram was generated using Circos [26]. D, Dinucleotide analysis of *M*. *hyorhinis* genome.

To reveal the functionality of mycoplasma MTases in the course of host cell infection, we determined the methylation pattern in the genomic DNA of planktonic versus intracellular *M*. *hyorhinis*. For these purposes, we analyzed a 368 bp CG-rich region of the *M*. *hyorhinis* genome by bisulfite sequencing that included two GATC and 27 CG sites. In all cultured bacteria, GATC sites were fully methylated (Fig 5A). As based on sequencing of individual colonies, half of the in vitro grown bacteria exhibited complete methylation of 27 CG sites. Remarkably, in approximately half of the cultured bacteria, CG methylation was limited.

We then asked whether a similar methylation pattern was maintained in the intracellular mycoplasma surviving long-term inside host cells. To answer this question, HTR8/SVneo trophoblasts were infected with *M*. *hyorhinis* and then infected trophoblasts were cultivated for 3 days in the presence of gentamicin to eliminate planktonic mycoplasma. Based on the subsequent methylation analysis of the 368 bp CG-rich region in the internalized, surviving mycoplasma, we observed that GATC sites remained completely methylated in the mycoplasma DNA while CG sites exhibited dramatic hypo-methylation (Fig 5B).

These data established that the GATC, but not CG, methylation pattern was evident and perhaps favorable for long-term infections. Conversely, the persistence of GATC methylation indicated that GATC-specific *Mhy*3 remained fully functional in internalized *M*. *hyorhinis*. Both the distribution and clustering of the CG and GATC potential methylation sites in the *M*. *hyorhinis* genome are illustrated in Fig 5C. The number of dinucleotide combinations in the *M*. *hyorhinis* genome is shown in Fig 5D.

## Discussion

*M*. *hyorhinis* was initially identified as a porcine parasite and a cause of polyarthritis and polyserositis in young pigs. However, *M*. *hyorhinis* constitutes an example of a possible emerging human pathogen. This versatile bacterium efficiently infects many human cell types [5, 12, 14, 16, 28]. We observed that invasive *M*. *hyorhinis* escaped endosomal degradation and autophagocytosis in infected human cells. Instead, according to our data, mycoplasma accumulated in exocysts, the membrane-tethered complexes involved in exocytosis. It appears that the association of *M*. *hyorhinis* with exocysts facilitates re-cycling of the bacteria and/or secreted factors to the extra-cellular environment. The earlier reports by others that mycoplasma proteins were found in the exosomes released by the infected tumor cells are consistent with our findings [28].

We demonstrated that both CG and GATC sites were hypermethylated in the free-living *M*. *hyorhinis*, potentially contributing to the mycoplasma survival in the host. In agreement, CG-specific DNA methylation in *Aspergillus fumigatus* DNA repressed Toll-like receptor-9 and pro-inflammatory cytokines in humans while unmethylated CG in *Aspergillus* DNA served as immune modulators and led to the activation of this Toll-like receptor and respective pro-inflammatory cytokines [29, 30].

Intriguingly, we also recorded the presence of “a low CG methylation” sub-population in free living *M*. *hyorhinis*. Following host cell invasion, CG-specific methylation became limited. It is possible that because of methylation-dependent silencing in the host, the high CG methylated mycoplasmas did not efficiently propagate in the infected cells. To this end, CG dinucleotides are already less frequent in mycoplasma relative to other bacteria. For example, in *M*. *hyorhinis* an average CG frequency is only 7.5 sites/1 kb ([31, 32] and Fig 5C and 5D). However, according to our genomic sequence analysis, there are ~50 segments in the *M*. *hyorhinis* genome in which CGs are similarly clustered compared to human CPGIs (Fig 5C).

It is likely that the variations in the CG methylation levels in *M*. *hyorhinis* genome contribute to the fitness and survival of this bacterium both inside and outside infected host cells. The most probable explanation is that the difference in the methylation patterns is a result of selection of a subset of mycoplasma that helps its survival inside host cells. Overall, our results and the data by others suggest that dynamic changes in the CG methylation pattern are important elements of the mycoplasma life cycle. Most probably, high levels of CG methylation contribute to the escape of the extracellular pathogen from the immune response, while CG hypomethylation, in addition to a low number of CG dinucleotides relative to other dinucleotide combinations in the *M*. *hyorhinis* genome (Fig 5D), is favorable for the pathogen survival inside the infected host cell [30].

From these perspectives, CG and GATC methylation of the pathogen genome may be reminiscent of the adenine methylation that is known to play an important role in patho-physiological processes in numerous Gram-negative bacteria, including viability and virulence [4]. We hypothesize that the dynamic changes in CpG methylation in mycoplasma trigger those gene expression changes which commit the bacteria to the intracellular versus the extracellular/adherent growth. Recently, gene regulation via the changes in dcm methylation was reported in *E*. *coli* [33, 34].

According to our resent observations [19], it is possible that active mycoplasma CG and GATC MTases released from the internalized mycoplasmas aberrantly methylate human genomic DNA and modify the human cell epigenome. By down-regulating its CG-specific methylation activity, the internalized mycoplasma can alleviate the potential impact of the counterproductive CG hyper-methylation on the host cell functions. Contrary, the effect of the GATC-specific methylation on the host cell genome is less severe [19].
